# A Fusion-Spliced Near-Field Optical Fiber Probe Using Photonic Crystal Fiber for Nanoscale Thermometry Based on Fluorescence-Lifetime Measurement of Quantum Dots

**DOI:** 10.3390/s110908358

**Published:** 2011-08-29

**Authors:** Takuro Fujii, Yoshihiro Taguchi, Toshiharu Saiki, Yuji Nagasaka

**Affiliations:** 1 School of Integrated Design Engineering, Keio University, Kanagawa 223-8522, Japan; 2 Department of System Design Engineering, Keio University, Kanagawa 223-8522, Japan; E-Mails: tag@sd.keio.ac.jp (Y.T.); nagasaka@sd.keio.ac.jp (Y.N.); 3 Department of Electronics and Electrical Engineering, Keio University, Kanagawa 223-8522, Japan; E-Mail: saiki@elec.keio.ac.jp

**Keywords:** near-field optics, photonic crystal fiber, quantum dots, thermometry

## Abstract

We have developed a novel nanoscale temperature-measurement method using fluorescence in the near-field called Fluorescence Near-field Optics Thermal Nanoscopy (Fluor-NOTN). Fluor-NOTN enables the temperature distributions of nanoscale materials to be measured *in vivo*/*in situ*. The proposed method measures temperature by detecting the temperature dependent fluorescence lifetimes of Cd/Se Quantum Dots (QDs). For a high-sensitivity temperature measurement, the auto-fluorescence generated from a fiber probe should be reduced. In order to decrease the noise, we have fabricated a novel near-field optical-fiber probe by fusion-splicing a photonic crystal fiber (PCF) and a conventional single-mode fiber (SMF). The validity of the novel fiber probe was assessed experimentally by evaluating the auto-fluorescence spectra of the PCF. Due to the decrease of auto-fluorescence, a six- to ten-fold increase of S/N in the near-field fluorescence lifetime detection was achieved with the newly fabricated fusion-spliced near-field optical fiber probe. Additionally, the near-field fluorescence lifetime of the quantum dots was successfully measured by the fabricated fusion-spliced near-field optical fiber probe at room temperature, and was estimated to be 10.0 ns.

## 1. Introduction

As highly integrated nano- and micro-scale devices have been developed over the last few decades, thermal design techniques at the nanoscale have become quite important. Various types of micro- and nanoscale thermometry techniques have been developed and improved. Among them, the contact-mode Scanning Thermal Microscopy (SThM) method using a thermocouple probe tip can achieve nanoscale spatial resolution; however, the heat transfer mechanism between the probe and the sample is still unverified [1,2]. Another contact-mode device, the Ga-filled carbon nano-thermometer, has the capability of measuring temperatures from room temperature to approximately 1,000 °C at the nano-scale [3,4]. However, this method has not been applied to actual temperature measurements because a proper method for handling the carbon nano-thermometer has not yet been established, and a Scanning Electron Microscope (SEM) is required to observe the changes in gallium in the nano-thermometer, which fluctuates according to temperature. Furthermore, generally in contact-mode methods, there is a concern that nano-structured samples may suffer damage due to the friction between the sample and the probe; also, the temperature change due to heat transfer during the operation can be significant.

An alternative optical nanoscale microscope technique, Near-field Scanning Optical Microscopy (NSOM), has been developed as a powerful tool for measuring optical phenomena with nanoscale spatial resolution beyond the diffraction limit of light. For example, Scattering-mode Near-field Scanning Optical Microscopy (S-NSOM) has been used to successfully measure the fluorescence lifetime of single nano-structured materials such as hetero-structured nano-rods with nanoscale spatial resolution [5]. However, the measurement is still performed under contact-mode conditions, because tapping-mode AFM is utilized in order to decrease the strong background noise. We have developed a non-contact and non-destructive nanoscale temperature measurement technique, namely Fluorescence Near-field Optics Thermal Nanoscopy (Fluor-NOTN) using local fluorescence lifetime detection of uniformly surface-modified Quantum Dots (QDs) [6]. In order to measure the local fluorescence lifetime of surface-modified quantum dots with nanoscale spatial resolution, Fluor-NOTN is based on an Illumination-Collection mode (I-C mode: both the illumination and the collection of the near-field light are operated in the proximity of the small aperture) NSOM technique. The surface-modified fluorescence probes are locally excited by near-field light, and the fluorescence lifetime, which contains information about the sample temperature, is then measured. QDs are utilized as the fluorescence probes due to certain useful characteristics such as their long fluorescence lifetime and high quantum yield.

In our previous publication, the fluorescence lifetimes of dried QD layers deposited on a Si substrate were successfully measured with probe apertures up to 100 nm, and relative temperature measurement by a nano-wire heater was accomplished [6]. However, the auto-fluorescence generated from the near-field optical fiber probe becomes a critical noise factor when the QDs are modified as a mono-molecular layer on the sample surface.

In this paper, we propose a novel low-auto-fluorescence fiber probe that is fabricated by fusion-splicing a Photonic Crystal Fiber (PCF) and a conventional Single Mode Fiber (SMF). Highly sensitive fluorescence lifetime measurement of QDs was achieved by using the newly fabricated probe. Here, the fabrication process of fusion-spliced near-field optical fiber probe is first demonstrated; then the resulting advance in near-field fluorescence detection of QDs using the fabricated probe is reported.

## 2. Method and Materials

### 2.1. Near-Field Optics

**Figure 1.**
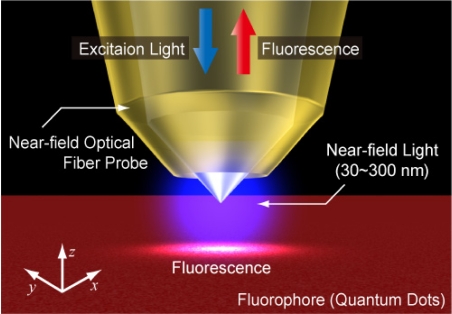
Schematic diagram of Fluor-NOTN. Quantum dots are utilized as temperature probes because of the temperature dependence of their fluorescence lifetime. Near-field light is utilized in order to achieve non-diffraction-limited spatial resolution.

In order to achieve nanoscale spatial resolution, the near-field light generated in the proximity of a small aperture was utilized. A schematic diagram of the Fluor-NOTN method is shown in Figure 1. In Fluor-NOTN, the excitation laser diode (wavelength: 473 nm) is coupled with an Au-coated near-field optical fiber probe. An excitation laser applies the electric field **E_0_** at the probe tip, and then the electrical dipole moment that interacts between the probe tip and the sample induces polarizability variation. The polarizability variation Δ*Ψ* can be expressed by the following equation [7]:

(1) $$\Delta \psi =\frac{\psi_{p}\psi_{s}}{2\pi {ɛ}_{0}r^{3}}$$

where *ψ_p_* and *ψ_s_* are the polarizability variation of the probe tip and the sample, respectively, *r* is the distance between the two, and *ε*_0_ is the vacuum electric permittivity. Thus, the intensity of the scattered light *I_s_* from the sample is expressed by the following equation [7]:

(2) $$\begin{array}{ll} I_{s} & \propto {\left|\left(\psi_{p}E_{0}+\Delta \psi E_{0}\right)+\left(\psi_{s}E_{0}+\Delta \psi E_{0}\right)\right|}^{2}\\ & \approx {\left(\psi_{p}+\psi_{s}\right)}^{2}{\left|E_{0}\right|}^{2}+4\Delta \psi \left(\psi_{p}+\psi_{s}\right){\left|E_{0}\right|}^{2}. \end{array}$$

The term that includes Δ*ψ*^2^ is regarded as negligible. The first term in Equation (2) indicates the propagation light element, which overlaps onto the signal as background noise. The second term indicates the scattered light element of the near-field light, and reflects the fact that the intensity of the near-field light is attenuated exponentially with the distance from the probe tip. Because the size of the near-field light depends merely on the aperture of the probe tip, but not the wavelength of the incident light, Fluor-NOTN enables the optical measurement of the temperature in nanoscale spatial resolution beyond the diffraction limit of light.

### 2.2. Fluorescence Lifetime Measurement

Fluor-NOTN utilizes a fluorophore such as QDs as temperature probes. In the fluorescence emission, the electrons of fluorophores are first excited to higher vibrational levels, and then transit to the ground state through a radiative or non-radiative process. The fluorescence lifetime, which is the average time that electrons stay in the excited state, is expressed by the following equation [8]:

(3) $$\tau =\frac{1}{W_{R}+W_{NR}}$$

where *W_R_* is the rate constant for radiative deactivation and *W_NR_* is that for non-radiative deactivation. *W_NR_* can be expressed by the following equation [8]:

(4) $$W_{NR}=s\;\text{exp}\frac{-\Delta U}{k_{B}T}$$

where *s* is the frequency factor, Δ*U* is the thermal energy of activation, *T* is the temperature, and *k_B_* is the Boltzmann constant. In the above equations, the rate of non-radiative process increases as the temperature increases; therefore, the fluorescence lifetime is expressed as a function of temperature, such that at higher temperatures the fluorophore has a shorter fluorescence lifetime. The fluorescence lifetime and its temperature dependence vary according to the type of fluorophore. In the case of QDs, it is known that the fluorescence lifetime is dependent on their diameter [9].

**Figure 2.**
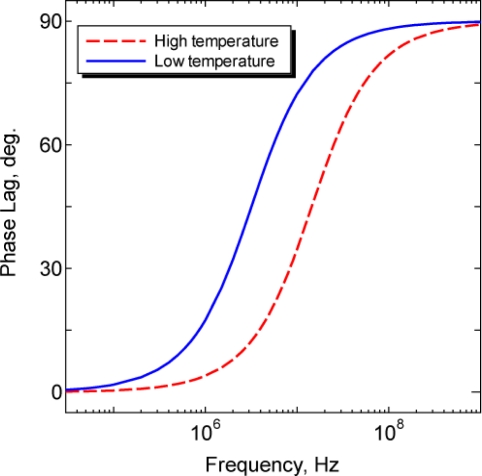
Simulated frequency-phase response in the frequency domain method. The phase lag increases as the modulating frequency increases due to the fluorescence lifetime. The solid and dashed curves show the same fluorophore at different temperatures; the frequency-phase response shifts to a higher frequency as the sample temperature increases.

In our research, the temperature dependence of the fluorescence lifetime is measured by the frequency domain method. In this method, a sinusoidally modulated laser is utilized as the excitation light. In this case, the excited fluorophores are forced to emit fluorescence at the same frequency. Due to the fluorescence lifetime, the fluorescence emission is delayed relative to the excitation, and the delay can be measured as the phase lag of excitation light and fluorescence. The fluorescence lifetime *τ* is expressed by the following equation:

(5) $$\tau =\frac{\text{tan}\varphi }{2\pi f}$$

where *f* is the modulation frequency and *ϕ* is the phase lag. The frequency-phase response derived by Equation (5) is shown in Figure 2. Owing to the temperature dependence of the fluorescence lifetime, the fluorescence lifetime becomes shorter as the sample temperature increases, indicating that the frequency-phase response shifts to the high-frequency region as shown in the figure.

### 2.3. Measurement Apparatus

**Figure 3.**
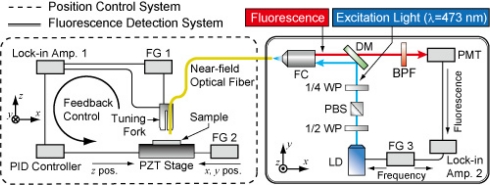
Experimental apparatus of Fluor-NOTN. WP, wavelength pass; PBS, polarized beam splitter; DM, dichroic mirror; FC, fiber coupler; PMT, photomultiplier tube; FG, function generator.

Figure 3 shows a schematic diagram of the Fluor-NOTN experimental apparatus. An excitation laser (LD, wavelength of 473 nm, modulation frequency of up to 250 MHz) is coupled into the near-field optical fiber and generates near-field light in the proximity region of the probe tip. Fluorophores on the sample surface are illuminated by the near-field light, and then the excited fluorescence is collected by the same fiber probe. The fluorescence is detected by the cooled photomultiplier tube (PMT) via a pin-hole and a band-pass filter. The phase lag of the detected fluorescent signal is measured by a lock-in amplifier. Qdot® 655/705 ITK™ Carboxyl Quantum Dots are utilized for the fluorescence probes. For the purpose of measuring the near-field fluorescence lifetime, QDs have some advantageous aspects such as: long fluorescence lifetime, high quantum yield, short half-bandwidth of emission spectrum, long Stoke’s Shift (indicating the separation between an absorption spectrum and an emission spectrum), ease of surface-modification/conjugating biomolecules, and being less prone to photobleaching. In our previous study, the fluorescence lifetime of Qdot655/705 at room temperature was measured to be 12∼17 ns and 22∼28 ns, respectively, by fluorescence microscope.

Since a near-field light is a nonpropagation light that is excited in the proximity region of the probe tip, the distance between the probe tip and the fluorophore should be controlled at the nanoscale (less than the size of the aperture). For the purpose of distance control, a near-field optical fiber was attached to the quartz crystal tuning fork and vibrated at its resonant frequency by a function generator. When the fiber approached the sample surface, the resonant frequency of the tuning fork changed due to the shear force (e.g., van der Waals force). Therefore, precise distance control was enabled by the detected amplitude or phase has some deviation then based on the feedback mechanism to maintaining the working point in the appropriate position on the resonance curve of the tuning fork.

### 2.4. Photonic Crystal Fiber

For the purpose of high-sensitivity temperature measurement, the weak fluorescence generated from the surface-modified QDs should be detected with a high S/N ratio. In our previous study, a custom-made Ge-doped silica core SMF was utilized to create a double-tapered near-field optical fiber probe that was optimized to I-C mode near-field operation using selective chemical etching of SMF [10]; however, the most critical noise was the auto-fluorescence generated from the dopant of SMF. Since the auto-fluorescence spectrum is broad, and the auto-fluorescence was simultaneously modulated at the same frequency of fluorescence emitted from QDs, the auto-fluorescence cannot be eliminated by a lock-in amplifier. In order to decrease this noise, we utilized a Photonic Crystal Fiber (PCF) for the near-field optical fiber probe. PCFs have a triangular pattern of micro air holes to form the cladding; therefore, PCFs usually consist of a single material (typically pure silica core and pure silica-air cladding). This aspect of PCFs is an important advantage for near-field fluorescence detection because the auto-fluorescence generated from the dopant is expected to be negligibly small.

**Figure 4.**
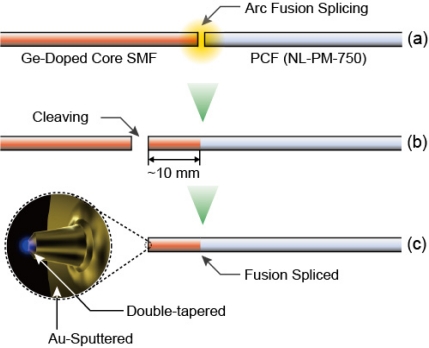
Fabrication of fusion-spliced near-field optical fiber for high-sensitivity near-field fluorescence detection. **(a)** The SMF and the PCF are fusion-spliced, **(b)** The spliced fiber is cleaved at the SMF side with the remaining length of the SMF up to 10 mm, in order to decrease the auto-fluorescence generated from the dopant of the SMF, **(c)** The double-tapered probe tip is then fabricated at the end face of the SMF side.

In order to use optical fibers as the near-field probe, selective chemical etching utilizing the different rates of solution between pure silica and doped silica is widely applied because of its precision and simplicity. However, this method cannot be applied to PCFs because of their silica-air cladding. In this paper, therefore, we propose a fusion-spliced near-field optical fiber probe fabricated by splicing a PCF and a conventional SMF, aiming at sensitive fluorescence detection from QDs. Figure 4 shows in brief the fabrication processes of the fusion-spliced near-field optical fiber. A PCF and a SMF are first fusion-spliced, and the spliced fiber is cleaved at the SMF side, with the remaining length of the SMF up to 10 mm. Selective chemical etching is then applied to the SMF side of the fusion-spliced fiber to fabricate the double-tapered core of the near-field optical fiber probe.

## 3. Results and Discussion

### 3.1. Probe Fabrication

**Figure 5.**
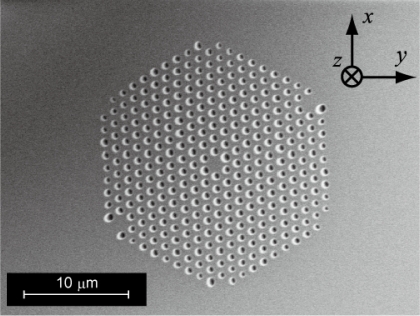
SEM images of the cross-section of NL-PM-750 consisting of a pure silica core and pure silica-air cladding.

As the materials for the fusion-spliced near-field optical fiber probe, we utilized NL-PM-750 (NKT Photonics Co.) as the PCF, and the Ge-doped SMF for the near-field fiber probe. An SEM image of the cross-section of NL-PM-750, which has a structure of a pure silica core and pure silica-air cladding, is shown in Figure 5. The fiber parameters are listed in Table 1. These two fibers are chosen because they have similar core diameters and Numerical Apertures (NAs); matching these parameters is one of the most important factors for decreasing the splice loss. Other important splicing parameters to decrease the splice loss caused by the collapse of micro air holes include: (1) fusion current; (2) fusion time; and (3) offset of splicing position [11]. In this work an average splice loss of 1.43 dB, and a minimum splice loss of 1.08 dB were achieved by optimizing these splicing parameters.

**Figure 6.**
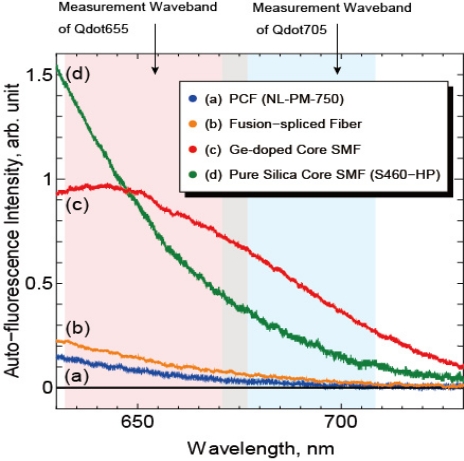
Auto-fluorescence spectra of **(a)** PCF (NL-PM-750, NKT Photonics Co.), **(b)** The fabricated fusion-spliced near-field optical fiber, **(c)** Ge-doped core SMF (JASCO Co.), and **(d)** Pure silica core and F-doped cladding SMF (S460-HP, Nufern Co.). The auto-fluorescence intensity of PCF is remarkably small compared to that of the conventional SMFs. In the case of the fusion-spliced fiber, an increase in auto-fluorescence caused by the spliced SMF was observed; however, the auto-fluorescence intensity was still much smaller than that of SMFs.

In order to evaluate the validity of the fusion-spliced fiber for near-field fluorescence detection, auto-fluorescence spectra of optical fibers were measured by spectroscope. Figure 6 shows the fluorescence spectra of NL-PM-750, the Ge-doped core SMF (JASCO Co.), a pure silica-core SMF with F-doped cladding (S460-HP, Nufern Co.), and the fusion-spliced fiber.

**Figure 7.**
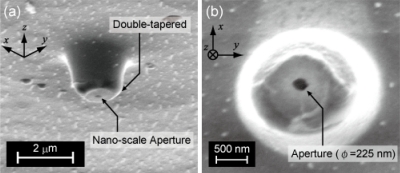
SEM images of the probe tip fabricated at the end of fusion-spliced near-field optical fiber. **(a)** A cross shot of the probe tip. The small aperture created by removing the Au coating and the shape of double-tapered core can be observed, **(b)** The vertical view. The diameter of the aperture is approximately 225 nm, smaller than the wavelength of the excitation (473 nm) and fluorescence of the QDs (655 nm).

All fibers were the same length (1.0 m), and the auto-fluorescence intensities were normalized by the intensities of the excitation laser coupled to the fibers. The auto-fluorescence intensity of the PCF at the measurement wavebands was much smaller than that of both the Ge-doped-core SMF and the pure-silica-core SMF as shown in Figure 6. The auto-fluorescence intensity of the fusion-spliced fiber was slightly stronger, approximately twice, than that of NL-PM-750 because of the spliced SMF whose length is about 6 mm. Compared with the SMFs, the auto-fluorescence intensities of the fusion-spliced fiber at the measurement waveband for Qdot655 and Qdot705 were reduced to 15.2% and 9.1%, respectively. Therefore, the fabricated fusion-spliced near-field optical fiber has the capability of detecting near-field fluorescence of QDs with an S/N ratio six to eleven times higher than that in the conventional near-field optical fiber probes. The fusion-spliced fiber is then fabricated to a fiber probe by chemical etching, Au-sputtering, and removal of the Au coating at the probe tip. An SEM image of the fabricated fusion-spliced near-field optical fiber probe is shown in Figures 7(a,b). The double-tapered probe tip was successfully fabricated, and a round-shaped aperture with a diameter of 225 nm, which is shorter than the wavelength of the excitation laser (473 nm), was observed.

### 3.2. Near-Field Fluorescence Detection

**Figure 8.**
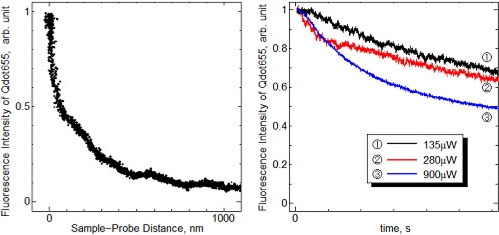
**(a)** The distance dependence of the near-field fluorescence intensity, **(b)** Change of the near-field fluorescence intensity by continuous exposure to the near-field light at the same height from the sample. Each curve was normalized to the initial intensity.

As a preliminary step for the temperature measurement using the fusion-spliced near-field optical fiber probe, the dried Qdot655 layers deposited on the Si substrate were utilized as the sample. Figure 8(a) shows the near-field fluorescence intensity of Qdot655 detected using the fabricated probe as a function of sample-probe distance.

The detected fluorescence intensity drastically increased in the proximity of the sample surface. A five-fold intensity change was observed with the vertical displacement of 350 nm, indicating the successful detection of near-field fluorescence of QDs, which can only be detected in the proximity of the sample as described in Equation (2). Figure 8(b) shows the time dependence of the near-field fluorescence intensity measured by the different excitation intensities. The detected near-field fluorescence intensity gradually decreased when the QDs were exposed by the near-field light, and the decrease rates of fluorescence were dependent on the intensity of the excitation light. This fluorescence reduction was attributed to photobleaching by continuous exposure to light [12].

**Figure 9.**
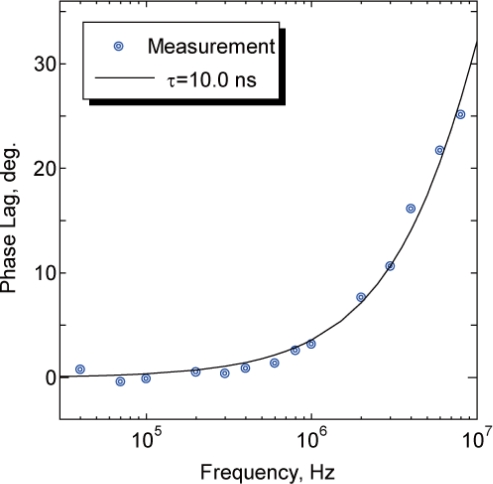
Phase-lag and fitting curve of Qdot655 measured by the fabricated probe. The fluorescence lifetime of Qdot655 at room temperature was calculated to be 10.0 ns.

We then measured the near-field fluorescence lifetime of Qdot655. Figure 9 shows the phase lag between the excitation laser and the near-field fluorescence of Qdot655 detected by the fabricated probe at room temperature. The phase lag measurement was carried out after the fluorescence intensity became stable to avoid the influence of photobleaching of QDs. The plots were fitted by the single-exponential decay model. The fluorescence lifetime of Qdot655 was estimated to be 10.0 ns, which is slightly shorter than the fluorescence lifetime estimated macroscopically using a fluorescence microscope in our previous study [7]. The shortening of the fluorescence lifetime may be due to the blue shift of QDs, which is the wavelength shift of the fluorescence caused by the photooxidation of fluorophores [13], or the quenching of QDs caused by the energy shift from the QDs to the metal probe [14]. Another factor, the difference in experimental conditions, especially the room temperature, is also possible.

The near-field fluorescence lifetime of QDs was successfully measured using a fusion-spliced near-field optical fiber probe fabricated by splicing a PCF and SMF, and the validity of local temperature measurement using QDs was evaluated. The excitation light will be decreased in our future study to avoid photooxidation and photobleaching of QDs.

## 4. Conclusions

We have proposed a novel nano-scale thermometry method based on the near-field fluorescence-lifetime measurement of Quantum Dots (QDs). In order to increase the sensitivity of the lifetime measurement, a Photonic Crystal Fiber (PCF) was utilized as the material for a low-auto-fluorescence fiber probe. The PCF and a conventional SMF having similar core diameters and NAs were fusion-spliced, and an average splice loss of 1.43 dB and minimum splice loss of 1.08 dB were achieved by minimizing the collapse of microscale air holes. By measuring the auto-fluorescence spectra of the SMFs, the PCF and the fabricated probe, it was validated that the auto-fluorescence intensity of the fabricated probe had been reduced to 15.2% at the measurement waveband of Qdot655, and 9.1% at the measurement waveband of Qdot705, respectively. We also measured the near-field fluorescence lifetime of dried Qdot655 layers using the fabricated probe with an aperture diameter of 225 nm. The near-field fluorescence lifetime of Qdot655 was successfully measured and was estimated to be 10.0 ns at room temperature. These measurement results verified the capability of the proposed fusion-spliced near-field optical fiber probe for highly sensitive near-field fluorescence sensing of QDs.

## Figures and Tables

**Table 1.** Parameters of the utilized fibers.

	**SMF (JASCO Co.)**	**NL-PM-750**
**Coating Diameter**	250 μm	240 ± 10 μm
**Cladding Diameter**	125 μm	120 ± 5 μm
**Core Diameter**	1.8 μm (estimated)	1.8 ± 0.3 μm
**Numerical Aperture (NA)**	0.32	0.38 ± 0.05 (at 780 nm)
**Cut-Off Wavelength**	760 nm	650 nm
**Core Material**	Ge-doped Silica	Pure Silica
**Cladding Material**	Pure Silica	Pure Silica and Air
